# Unusual metastasis of papillary thyroid cancer to the pancreas, liver, and diaphragm: a case report with review of literature

**DOI:** 10.1186/s12893-020-00748-1

**Published:** 2020-04-22

**Authors:** Haoyuan Ren, Nengwen Ke, Chunlu Tan, Xing Wang, Wen Cao, Xubao Liu

**Affiliations:** 1 Department of Pancreatic Surgery, West China Hospital, Sichuan University, GuoXue Lane No 37, Chengdu, 610041 China; 2 The People’s Hospital of Deyang, Deyang, 618000 Sichuan China

**Keywords:** Papillary thyroid cancer, Pancreas, Metastasis, Pancreaticoduodenectomy

## Abstract

**Background:**

Papillary thyroid cancer (PTC) is the most common form of well-differentiated endocrine malignancy. Distant metastases of PTC are rare and usually occur in the bones, lungs, and thoracic lymph nodes despite the common locoregional metastases to the lymph nodes of the neck. The metastasis of PTC to the pancreas is extremely rare. Here, we present a patient with PTC that had simultaneously metastasized to the pancreas, liver, and diaphragm.

**Case presentation:**

A 47-year-old male patient suffering from mild abdominal pain for 2 months was admitted to our hospital. The ultrasound (US) and computed tomography (CT) scan of the abdomen revealed a pancreatic space-occupying lesion and pancreatic duct dilatation, and the patient underwent exploratory laparotomy. Intraoperative examination identified a hard mass (approximately 4.0 cm × 3.0 cm) in the body and tail of the pancreas and a mass (1.5 cm in diameter) in the diaphragm. Three light masses were also noted on the surface of his liver. The patient underwent radical distal pancreatectomy, splenectomy, diaphragm, and liver mass resection. After surgery, the pathological report revealed that the masses resected from the pancreas, liver, and diaphragm were PTC metastases. Then, the patient had a thyroid US and an endoscopic US-guided fine needle aspiration biopsy of the thyroid mass. Pathology showed papillary cancer. Subsequently, the patient received a complete thyroidectomy, a cervical lymphadenectomy, bilateral parotidectomy, and bilateral submandibular gland resection.

**Conclusions:**

Aggressive surgeries, such as pancreaticoduodenectomy (PD), should be considered for selected patients with metastatic diseases from PTC to alleviate the symptoms and prolong their survival.

## Background

Papillary thyroid cancer (PTC) is the most common form of well-differentiated endocrine malignancy [1]. The main manifestation of PTC is a neck mass and a thyroid nodule. Distant metastases of PTC are rare and usually occur in the bones, lungs, and thoracic lymph nodes despite the common locoregional metastases to the lymph nodes of the neck [2–4]. PTC metastasize to the pancreas are extremely rare. To date, only 12 cases have been reported in literature, and only one of these cases is the first clinical manifestation due to metastasis [5–15]. Here, we present a patient with PTC that had simultaneously metastasized to the pancreas, liver, and diaphragm from our institution. The metastasis in the pancreas caused his first clinical manifestations which mimicked the primary pancreatic cancer. This rare case has never been reported previously.

## Case presentation

**Fig. 1:**
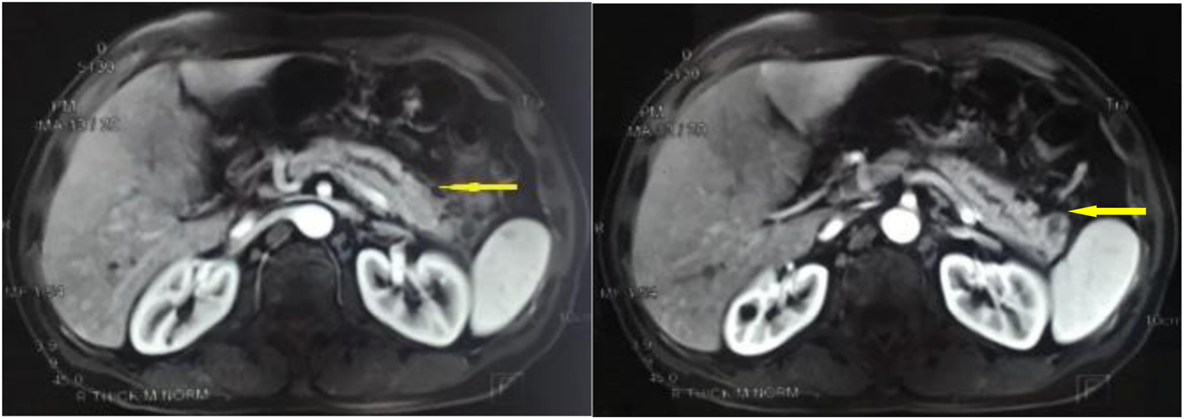
CT scan of the abdomen revealed a pancreatic space-occupying lesion and pancreatic duct dilatation

**Fig. 2:**
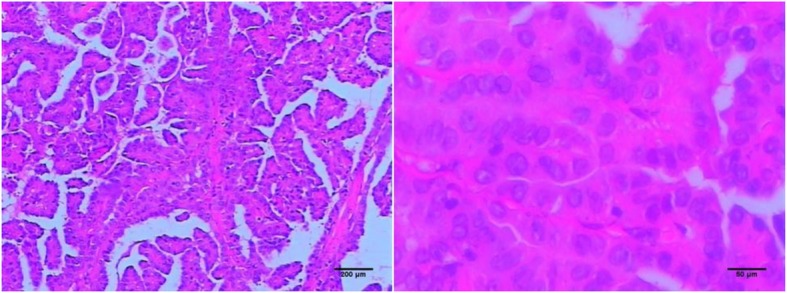
Pathology showed the pancreatic metastasis of PTC

**Fig. 3:**
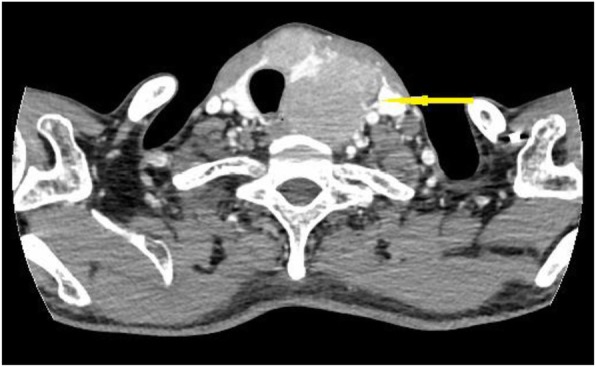
CT scan showed large masses in the isthmus and left lobes of the thyroid

**Fig. 4:**
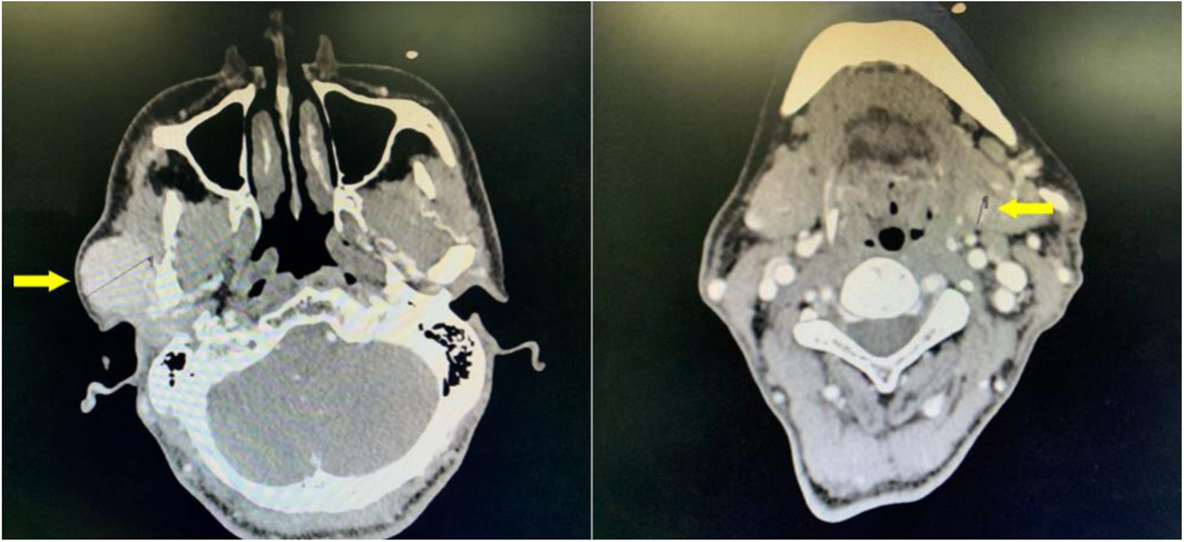
CT scan showed multiple masses in the bilateral parotid and submandibular gland

A 47-year-old male patient suffering from mild abdominal pain for 2 months was admitted to our hospital in February 2018. He was diagnosed with “acute pancreatitis” first before transferring to our department. The ultrasound (US) and computed tomography (CT) scan of the abdomen revealed a pancreatic space-occupying lesion and pancreatic duct dilatation (Fig. 1). The serum amylase and lipase levels were slightly elevated (231 and 546 U/L, respectively; normal range: 25–125 and 13–60 IU/L, respectively). the preoperative serum CA 19–9 level was 34.82 U/ml. Then, the patient underwent exploratory laparotomy. Intraoperative examination identified a hard mass in the body (approximately 4.0 cm × 3.0 cm) and tail of the pancreas, varicose veins around the spleen, a mass in the diaphragm (1.5 cm diameter), and three light masses on the surface of the liver. One mass was taken for pathological examination of the intraoperative rapid frozen section, and the result showed adenocarcinoma in the mass. The patient underwent radical distal pancreatectomy, splenectomy, diaphragm, and liver mass resection. The patient manifested with obstructive jaundice after surgery and gradually increased level of bilirubin. The total bilirubin increased from 65.4 μmol/L to 105.6 μmol/L and then to 140.1 μmol/L, and the direct bilirubin increased from 53.8 μmol/L to 81.0 μmol/L and then to 118.1 μmol/L. Subsequently, the patient underwent cholangiojejunostomy, and the pathological report revealed resected masses from the pancreas, liver, and diaphragm, indicating PTC metastases (Fig. 2). Immunohistochemical studies showed positive stanning of TG(+), PAX-8(+), TTF-1(+), CK19(+), HBME-1(+), Galectin-3(+), P53(+), WT(+), DPC4(+), CA19–9(luminal surface+), MUC1(+), with negative staining of MUC5AC(−), MUC6(−), MUC2(−). Then, the patient had a thyroid US, which showed multiple hypoechoic masses in the left thyroid gland and an endoscopic US-guided fine needle aspiration (FNA) biopsy of the thyroid mass. Pathology also revealed papillary cancer. After the patient had recovered in the pancreatic department, he was transferred to the thyroid department. A CT scan was taken, and the result showed large masses in the isthmus and left lobes of the thyroid, multiple enlarged lymph nodes, and multiple masses in the bilateral parotid and submandibular gland (Figs.3 and 4). Then, the patient received an FNA biopsy of the parotid and submandibular mass, and the result showed PTC metastases. Immunohistochemical studies showed TTF-1(+), TG (−), CK19(+), HBME-1(+), Galectin-3(+), Villin(−), CDX-2(−). In July 2018, he received complete thyroidectomy, cervical lymphadenectomy, bilateral parotidectomy, bilateral submandibular gland and left recurrent laryngeal nerve resection. Intraoperative examination showed a huge irregular mass (approximately 10.0 cm × 7.0 cm × 5.0 cm) in the left and isthmus of the thyroid gland with calcification. The mass invaded the left recurrent laryngeal nerve and adhered to the surface of the trachea, and enlarged lymph nodes (3.5 and 2.5 cm in diameter) were found in the bilateral parotid. Multiple small enlarged lymph nodes (diameter ranging from 0.3 cm to 1.5 cm) were also noted in the bilateral submandibular gland. Intraoperative rapid frozen biopsy showed papillary cancer. The final histopathology revealed bilateral thyroid and isthmic papillary carcinoma and cervical lymph node metastasis, and papillary cancer in the left parotid and bilateral submandibular glands but not in the right parotid gland. Immunohistochemical studies showed CK19(+), HBME-1(+), Galectin-3(+), TG (partial+), TTF-1(+), P53(partial+), NapsinA (−), PD-L1(+, approximately 10%). The gene test showed activated mutation detected in the exon 15 of the BRAF gene (V600E) and the promoter 228 of TERT. After surgery, the patient was given radioiodine-131 therapy. He recovered well and was discharged from the hospital with oral Euthyrox therapy. The patient still survives at present.

## Discussion and conclusion

PTC has no early typical symptoms due to its anatomic location. Therefore, a delay in clinical diagnosis is inevitable, which leads to the advanced stage of this neoplasm. Thus, the primary tumor in the thyroid is always not diagnosed until the symptoms of secondary metastatic tumor appear. Pancreatic metastases also remain asymptomatic for a long time before diagnosis or manifest pancreatitis symptoms, as seen in our patient. The first and main manifestation of this patient is abdominal pain for 2 months. If a patient does not have thyroid examination and has no history of thyroid disease, the primary thyroid cancer is always ignored. Conventional inspection tools (CT and US) may fail to distinguish the type of tumor in the pancreas. Positron emission tomography–CT can detect the primary neoplasm of the body and pancreatic lesions sensitively, but it has not been performed on our patient because the diagnosis of the pancreatic neoplasm is primary.

Whether the pancreatic metastatic tumor surgery is beneficial to the prognosis of patients is disputed because of the high rate of morbidity and mortality after pancreatectomy. We have reviewed the previous cases and performed surgery when the metastases are limited and causing symptoms. The primary tumor is resected first, followed by the metastatic tumor. This procedure relieves the symptoms and may increase the survival of patients. The selection criteria for surgery may be that the primary tumor has good prognosis, and the primary tumor can be resected. The metastasis is also isolated to the pancreas, and the patient can tolerate pancreatectomy [5]. Our patient has undergone radical distal pancreatectomy, splenectomy, and diaphragm and liver mass resections after laparotomy exploration. Then, the primary thyroid cancer and the local metastases are resected. However, if the primary thyroid is diagnosed first, and several metastatic sites are found, whether the subsequent surgery like pancreatectomy should be conducted is disputed. Moreover, whether metastatic and primary tumor resections can be taken simultaneously remains questionable because of increased risk. The strategy of treatment and surgery should be formulated under the discussion of a multidisciplinary team. Adjunct and multikinase inhibitors (sorafenib or sunitinib) may also slow down the disease progression, especially in patients with BRAF V600E mutation in tumor cell lines [16].

In conclusion, from this case report and from the few previous cases documented in literature, aggressive surgeries, such as pancreaticoduodenectomy, should be considered in selected patients with metastatic disease from PTC to alleviate the symptoms and prolong the survival.

## Data Availability

All data generated or analysed during this study are included in this published article.

## Abbreviations

PTC: Papillary thyroid cancer
CT: Computed tomography
US: Ultrasound
FNA: Fine needle aspiration
PD: Pancreaticoduodenectomy

## References

[1] Jemal A, Siegel R, Xu J, Ward E (2010). Cancer statistics. CA Cancer J Clin.

[2] Mazzaferri EL, Jhiang SM (1994). Long-term impact of initial surgical and medical therapy on papillary and follicular thyroid cancer. Am J Med.

[3] Haigh PI, Urbach DR, Rotstein LE (2005). Extent of thyroidectomy is not a major determinant of survival in low- or high-risk papillary thyroid cancer. Ann Surg Oncol.

[4] Sampson E, Brierley JD, Le LW, Rotstein L, Tsang RW (2007). Clinical management and outcome of papillary and follicular (differentiated) thyroid cancer presenting with distant metastasis at diagnosis. Cancer.

[5] Li XO, Li ZP, Wang P (2014). Pancreatic metastasis of papillary thyroid carcinoma: a case report with review of the literature. Int J Clin Exp Pathol.

[6] Sugimura H, Tamura S, Kodama T (1991). Metastatic pancreas cancer from the thyroid; clinical imaging mimicking non functioning islet cell tumor. Radiat Med.

[7] Jobran R, Baloch ZW, Aviles V, Rosato EF, Schwartz S, LiVolsi VA (2000). Tall cell papillary carcinoma of the thyroid: metastatic to the pancreas. Thyroid..

[8] Meyer A, Behrend M (2006). Is pancreatic resection justified for metastasis of papillary thyroid cancer?. Anticancer Res.

[9] Siddiqui AA, Olansky L, Sawh RN (2006). Pancreatic metastasis of tall cell variant of papillary thyroid carcinoma: diagnosis by endoscopic ultrasound-guided fine needle aspiration. JOP.

[10] Angeles-Angeles A, Chable-Montero F, Martinez-Benitez B, Albores-Saavedra J (2009). Unusual metastases of papillary thyroid carcinoma: report of 2 cases. Ann Diagn Pathol.

[11] Borschitz T, Eichhorn W, Fottner C (2010). Diagnosis and treatment of pancreatic metastases of a papillary thyroid carcinoma. Thyroid.

[12] Chen L, Brainard JA (2010). Pancreatic metastasis from papillary thyroid carcinoma diagnosed by endoscopic ultrasound-guided fine needle aspiration: a case report. Acta Cytol.

[13] Alzahrani AS, AlQaraawi A, Al Sohaibani F, Almanea H, Abalkhail H (2012). Pancreatic metastasis arising from a BRAF(V600E)-positive papillary thyroid cancer: the role of endoscopic ultrasound-guided biopsy and response to sorafenib therapy. Thyroid..

[14] Tunio MA, Alasiri M, Riaz K, Alshakweer W (2013). Pancreas as delayed site of metastasis from papillary thyroid carcinoma. Case Rep Gastrointest Med.

[15] Davidson M, Olsen RJ, Ewton AA, Robbins RJ (2017). Pancreas Metastases From Papillary Thyroid Carcinoma: A review of the literature. Endocr Pract.

[16] Kimura ET, Nikiforova MN, Zhu Z (2003). High prevalence of BRAF mutations in thyroid cancer: genetic evidence for constitutive activation of the RET/PTC-RAS-BRAF signaling pathway in papillary thyroid carcinoma. Cancer Res.

